# Supplementary material for: Erg6 Acts as a Downstream Effector of the Transcription Factor Flo8 To Regulate Biofilm Formation in Candida albicans
Source: Microbiol Spectr. 2023 Apr 26;11(3):e00393-23. doi: 10.1128/spectrum.00393-23 (PMC10269489; doi:10.1128/spectrum.00393-23)
Supplement: Supplemental file 1 — Supplemental material and methods, Fig. S1 and S2, and Tables S1 to S3. Download spectrum.00393-23-s0001.pdf, PDF file, 0.5 MB [file spectrum.00393-23-s0001.pdf]

Supplemental Material

Erg6 acts as a downstream effector of the transcription factor Flo8 to regulate biofilm formation in *Candida albicans*

Xueyang Jin,[a#] Xiaoyi Luan,[a#] Fei Xie,[b] Wenqiang Chang,[a*] Hongxiang Lou[a*]

[a]Department of Natural Product Chemistry, Key Laboratory of Chemical Biology (Ministry of Education), School of Pharmaceutical Sciences, Cheeloo College of Medicine, Shandong University, Jinan, Shandong Province, China.

[b]Department of Pharmacy, Qilu Hospital of Shandong University, Jinan, Shandong Province, China.

[#] These authors contributed equally to this work.

[*] Correspondence: louhongxiang@sdu.edu.cn or changwenqiang@sdu.edu.cn

Materials and methods

Strains and growth conditions

The strains used in this study are shown in **Table S1**. Cells were routinely pre-grown overnight at 30 °C in liquid YPD medium (1% yeast extract, 2% peptone, 2% glucose). For experiments, 5 mL of YPD broth was inoculated with a single colony of *C. albicans*, followed by incubation overnight at 30 °C and 200 rpm orbital shaking. The next day, the yeast cells were harvested by centrifugation, washed three times with PBS (pH 7.4), and adjusted to the concentration required for the subsequent experiment.

Strain construction

Spontaneous Ura^- derivatives (*flo8Δ/Δ-ura3*) were generated in strain *flo8Δ/Δ* through using 5-fluoro-orotic acid-containing medium. pBA1-*ERG6* for the overexpression of *C. albicans ERG6* in *C. albicans* was constructed by placing a 1131-b PCR fragment containing *ERG6* coding sequence into the *ApaI* site of plasmid pBA1 (1). Primers used for PCR amplification were shown in **Table S2**. The plasmid pBA1-*ERG6* was linearized by digestion with *AcsI* and used to transform *flo8Δ/Δ-ura3* by the lithium acetate method (2). For generating *flo8Δ/Δ*+pBA1 strain in *flo8Δ/Δ-ura3*, the plasmid pBA1 was linearized by digestion with *AcsI* and used to transform *flo8Δ/Δ-ura3* by the lithium acetate method.

Biofilm formation

C. albicans cells were prepared in RPMI1640 medium at a cell density of 1×10^6 cells/mL. The inoculated plate was incubated at 37 °C for 90 min for initial adhesion of cells. The wells of microtiter plates were washed with PBS, and 100 μL of fresh

RPMI1640 medium was added. The plate was incubated at 37 °C for an additional 6, 12, 24 or 48 h to allow biofilm formation. Then the medium was discarded, and each well was washed three times with PBS to remove non-adherent cells. Biofilm formation was observed under an Olympus microscope (Olympus IX71, Japan) and quantitatively analyzed by the XTT reduction assay (3).

Morphological transition test

C. albicans cell suspensions of 1×10^4 cells/mL were prepared in RPMI1640 medium in 96-well microplates and incubated for 6 h at 37 °C. Then, images were acquired by a microscope.

Sterol composition analysis

C. albicans cells (1×10^6 cells/mL) were inoculated into 200 mL of Mops-buffered RPMI1640 medium and cultured with shaking for 12 or 24 h at 37 °C. The cells were harvested by centrifugation, quickly washed with deionized water, and lyophilized for sterol extraction.

Saponification and extraction of 50.0 mg of dried cells were carried out in 6 mL of pyrolysis solution (25.0 g of sodium hydroxide, 35.0 mL of distilled water, 100.0 mL of absolute ethyl alcohol) for 2 h at 85 °C. After cooling to room temperature, 0.5 mg of *β*-sitosterol (Meilunbio, China) was added as an internal standard, and the total intracellular sterols were extracted with petroleum ether. The crude extract was concentrated under reduced pressure at 40 °C and diluted with chromatographically pure hexane (TEDIA, USA), and sterol analysis was performed by chromatography–mass spectrometry (GC–MS) on a Prominence nano-LTQ Orbitrap velos pro ETD. The

ionization mode was electron impact (EI) with a 70-eV electron beam. The samples were detected on a TG-5 column (30 m × 0.25 μm × 0.25 μm, Thermo Scientific) coated with 5% phenyl-methyl polysiloxane as the stationary phase. Ultrapure helium was used as the carrier gas at a constant flow rate of 1 mL/min. One microliter of the sample was injected in split mode with a split ratio of 1:10. For MS, a delay of 3 min and a range of 50–650 Da were used. The injector temperature was 250 °C, and the quadrupole temperature was 300 °C. The temperature of the GC oven was initially 60 °C and was ramped up to 290 °C at 10 °C/min, followed by a hold for 5 min. Metabolites were identified by reference to the NIST17 database.

qPCR analysis

Quantitative real-time PCR (qPCR) was performed to measure the transcriptional expression of several genes involved in ergosterol synthesis and biofilm formation. The assayed genes and the corresponding primers are indicated in **Table S3**. Total RNA was isolated using the hot phenol method and converted to cDNA as previously described (4). Then the SYBR Green-based PCR assays were carried out in an Eppendorf Real-Time PCR System. *ACT1* served as the internal control.

***In vivo* evaluation using a *G. mellonella* infection model**

G. mellonella larvae were used as an *in vivo* model to evaluate the virulence of different *C. albicans* strains (5). Larvae with a body weight of approximately 0.25 g and without melanization were randomly divided into five groups (*flo8Δ/Δ*, *flo8Δ/Δ*+pBA1, *flo8Δ/Δ*+pBA1-*FLO8*, *flo8Δ/Δ*+pBA1-*ERG6* and vehicle group). Cells of each *C. albicans* strain were diluted to 2×10^7 CFU/mL with PBS, and 20 larvae per

group were each injected with 10 μL of *C. albicans* suspension via the last right proleg. The blank group was injected with the same volume of PBS. The larvae were cultured at 30 °C, and survival was recorded daily for 7 days.

Another four groups (each containing 9 larvae) treated as described above were used to assess the fungal burden of the infected larvae. On the fourth day, the surface of each larva was disinfected with 75% ethanol. Then, each larva was homogenized in 1 mL of sterile PBS by vortexing with glass beads. *Candida* cell counts were performed by smearing serial dilutions of the homogenate onto YPD plates. The surviving cells in each group were calculated by enumeration of colonies after 48 h of growth at 30 °C. For histopathological analysis, 50 larvae were divided into 5 groups and treated as described above. At 48 h post-infection, two larvae were randomly selected from each group and fixed in 4% paraformaldehyde. The larvae were embedded in paraffin wax, sectioned and subjected to periodic acid-Schiff (PAS) staining. The tissue slides were examined under an Olympus microscope at 40× magnification.

Yeast one-hybrid assay

For the yeast one-hybrid (Y1H) screening assay, the promoter of *ERG6* was synthesized in two parts by Zoonbio Biotechnology Co. Ltd and cloned into the pHIS2 vector. The full-length encoding region of *FLO8* was ligated into the pGADT7 vector. The recombinant pHIS2-*ERG6*promoter-Apart (-426 bp to -942 bp) or pHIS2-*ERG6*promoter-Bpart (-1 bp to -427 bp) was linearized and used to transform *S. cerevisiae* Y187 for the self-activation test. The results suggested that *S. cerevisiae* Y187 transformed with pHIS2-*ERG6*promoter-Bpart showed strong self-

activating activity (Fig S1). Then, the recombinant pHIS2-*ERG6*promoter-Apart and empty pGADT7 or pGADT7-Flo8 were linearized and used to transform *S. cerevisiae* Y187. The transformed yeast cells were screened on SD/−Leu-Trp medium and SD/−Leu-Trp-His + 10 mM 3-amino-1,2,4-triazole (3-AT) medium.

Statistical analysis.

The statistical significance of differences between treated and control groups was evaluated by one-way ANOVA test (total number of groups > 2) or Student's *t*-test (total number of groups = 2). For survival analysis, the long-rank (Mantel-Cox) test was used to compare differences between groups. Data were showed as mean ± standard deviations (SDs). Statistical significance was determined according to the P value. $^*P < 0.05$, $^{**}P < 0.01$, $^{***}P < 0.001$.

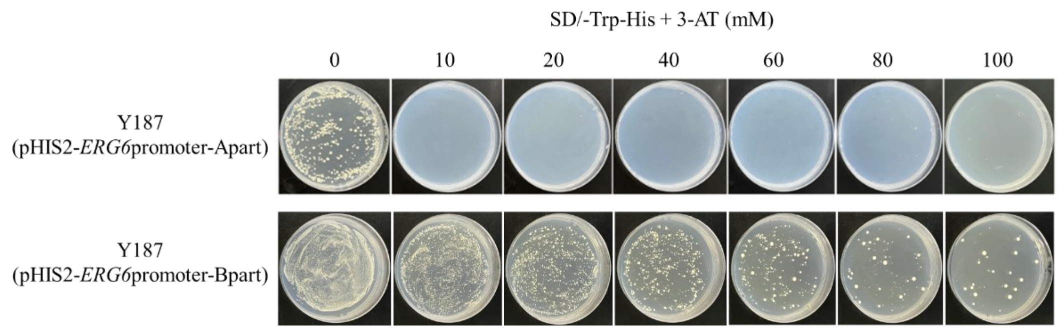

FIG S1 Growth of *S. cerevisiae* Y187 (containing pHIS2-*ERG6*promoter-Apart) or Y187 (containing pHIS2-*ERG6*promoter-Bpart) at different concentrations of 3-AT. *S. cerevisiae* cells were spread on a SD agar plate containing different concentrations of 3-AT. Images were acquired after 3 d incubation at 30 °C

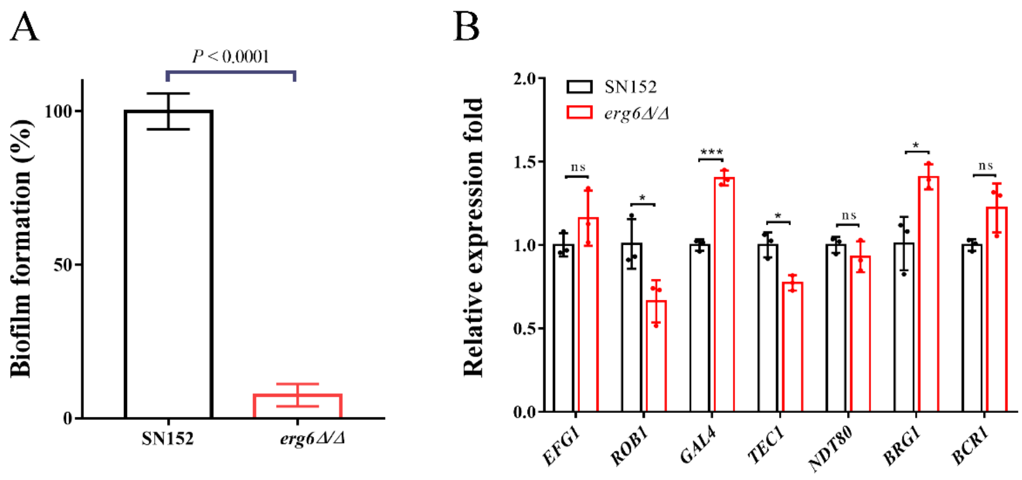

FIG S2 Deletion of *ERG6* leads to defects in *C. albicans* biofilm formation. (A) 1×10^6 *C. albicans* SN152 or *erg6Δ/Δ* cells were incubated in RPMI1640 medium for 24 h at 37 °C. Then the cells in the wells were washed three times with PBS. The biofilm formation was quantitatively assessed using the XTT reduction assay. The bars represent means ± SDs. (B) 1×10^6 *C. albicans* SN152 or *erg6Δ/Δ* cells were incubated in RPMI1640 medium for 24 h at 37 °C. The transcription levels of genes associated with biofilm formation in *erg6Δ/Δ* as determined by PCR are indicated as the fold change relative to SN152. The bars represent the means ± SDs from three independent experiments. *$P < 0.05$, **$P < 0.01$, ***$P < 0.001$.

Table S1. Strains used in this study

Strain	Phenotype	Source
flo8Δ/Δ	*ura3::imm434/ura3:: imm434 flo8::hisG/flo8::hisG-URA3-hisG*	(1)
flo8Δ/Δ-ura3	*ura3::imm434/ura3:: imm434 flo8::hisG/flo8::hisG*	This study
flo8Δ/Δ+pBA1	*ura3::imm434/ura3:: imm434 flo8::hisG/flo8::hisG-URA3-hisG ADE2/ade2::ADH1p-URA3*	This study
flo8Δ/Δ+pBA1-*FLO8*	*ura3::imm434/ura3:: imm434 flo8::hisG/flo8::hisG-URA3-hisG ADE2/ade2::ADH1p-FLO8-URA3*	(1)
flo8Δ/Δ+pBA1-*ERG6*	*ura3::imm434/ura3:: imm434 flo8::hisG/flo8::hisG-URA3-hisG ADE2/ade2::ADH1p-ERG6-URA3*	This study
SN152	*arg4Δ/arg4Δ leu2Δ/leu2Δ his1Δ/his1Δ URA3/ura3Δ::imm434 IRO1/iro1Δ ::imm434*	Lab stock
erg6Δ/Δ	*erg6Δ::HIS1/erg6Δ::LEU2 arg4 Δ /arg4Δ URA3/ura3Δ::imm434 IRO1/iro1Δ ::imm434*	Lab stock

Table S2. Primers used in this study

Primers	Sequence[a]	Application
pBA1-*ERG6*-F	gtcgacctcgagggggggcccATGTCTCCAGTTCAATTAGCAGAAAAAAATTACGA	gene ORF region of *ERG6* for overexpression
pBA1-*ERG6*-R	ggcgaattgggtaccgggcccTTAATCTTTCTTTTCTAATGGTTTTCTAACAACGTA	

[a] Fragments in lowercase letters are restriction sites

Table S3. Primers used for quantitative real-time PCR

Primers	Sequence
ERG6-F	ACAAGCTACTGCTAGACAT
ERG6-R	ATCTTGTGATTTCTCTACCAG
EFG1-F	TATGCCCCAGCAAACAACTG
EFG1-R	TTGTTGTCCTGCTGTCTGTC
BRG1-F	ACTTTCTTCACCTACATTGC
BRG1-R	TGGTGAAGATTGTTGTTGGT
TEC1-F	AGGTTCCCTGGTTTAAGTG
TEC1-R	ACTGGTATGTGTGGGTGAT
ROB1-F	GCCCACAAGAATGATTTAGCAAC
ROB1-R	CCAGATAATTCCCATGCAGTAGT
GAL4-F	CACCTACCCTTTACGCCAGTC
GAL4-R	GGTGGTGGTGGTGATGTTGTTTA
NDT80-F	ATGCCAGTGACACCATTGTTGC
NDT80-R	GCTAGGGTGATGAGGTGGGAAA
BCR1-F	CAACACTAACGCCGACATT
BCR1-R	TACAACCAGGATATCCAGTA
ACT1-F	TCCAGAAGCTTTGTTCAGAC
ACT1-R	TGCATACGTTCAGCAATACC

References

1. Cao F, Lane S, Raniga PP, Lu Y, Zhou Z, Ramon K, Chen J, Liu H. 2006. The Flo8 transcription factor is essential for hyphal development and virulence in *Candida albicans*. *Mol Biol Cell* 17:295-307.
2. Zeng G, Wang Y-M, Chan FY, Wang Y. 2014. One-step targeted gene deletion in *Candida albicans* haploids. *Nat Protoc* 9:464-473.
3. Chang W, Liu J, Zhang M, Shi H, Zheng S, Jin X, Gao Y, Wang S, Ji A, Lou H. 2018. Efflux pump-mediated resistance to antifungal compounds can be prevented by conjugation with triphenylphosphonium cation. *Nat Commun* 9:5102.
4. Zhang M, Chang W, Shi H, Zhou Y, Zheng S, Li Y, Li L, Lou H. 2017. Biatriosporin D displays anti-virulence activity through decreasing the intracellular cAMP levels. *Toxicol Appl Pharmacol* 322:104-112.
5. Champion OL, Titball RW, Bates S. 2018. Standardization of *G. mellonella* larvae to provide reliable and reproducible results in the study of fungal pathogens. *J Fungi (Basel)* 4:108.
